# Profiling the expression domains of a rice-specific microRNA under stress

**DOI:** 10.3389/fpls.2015.00333

**Published:** 2015-05-13

**Authors:** Neha Sharma, Anita Tripathi, Neeti Sanan-Mishra

**Affiliations:** Plant Molecular Biology Group, International Centre for Genetic Engineering and Biotechnology, New Delhi, India

**Keywords:** rice, salt stress, microRNA, expression patterns, panicles

## Abstract

Plant microRNAs (miRs) have emerged as important regulators of gene expression under normal as well as stressful environments. Rice is an important cereal crop whose productivity is compromised due to various abiotic stress factors such as salt, heat and drought. In the present study, we have investigated the role of rice-specific Osa-miR820, in indica rice cultivars showing contrasting response to salt stress. The dissection of expression patterns indicated that the miR is present in all the tissues but is enriched in the anther tissues. In salinity, the miR levels are up-regulated in the leaf tissues but down-regulated in the root tissues. To map the deregulation under salt stress comprehensive time kinetics of expression was performed in the leaf and root tissues. The reproductive stages were also analyzed under salt stress. It emerged that a common regulatory scheme for Osa-miR820 expression is present in the salt-susceptible Pusa Basmati 1 and salt-tolerant Pokkali varieties, although there is a variation in the levels of the miR and its target transcript, OsDRM2. The regulation of Osa-miR820 and its target were also studied under other abiotic stresses. This study thus captures the window for the miR-target correlation and the putative role of this regulation is discussed. This will help in gaining useful insights on the role of species specific miRs in plant development and abiotic stress response.

## Introduction

The sessile nature of plants necessitates a strict and rapid monitoring of gene expression as an important aspect of proper growth and development, thereby delineating a crucial role for the regulatory molecules. MicroRNAs (miRs) are a newly identified class of endogenous non-coding RNA molecules that play a major role in genome dynamics at the molecular level (Voinnet, 2009). The miRs regulate target mRNA expression in a sequence-specific manner, by its cleavage or by translational repression (Llave et al., 2002; Aukerman and Sakai, 2003; Addo-Quaye et al., 2008; Brodersen et al., 2008; Rogers and Chen, 2013). Increasing evidence supports the fact that miRs act as genetic buffers in plant gene regulation providing protection against various abiotic and biotic stress conditions (Sunkar et al., 2007; Sanan-Mishra et al., 2009; Khraiwesh et al., 2012). It is thus important to understand the mechanism of miR mediated regulation of the genetic machinery in plants, under hostile conditions.

The first direct evidence for the involvement of miRs in plant stress responses came from the work of Bartel’s group in *Arabidopsis*, where they identified several novel miRs, including miR395, which were up-regulated upon sulfate starvation (Jones-Rhoades and Bartel, 2004). Ath-miR395 targets a low-affinity sulfate transporter, AST68 and three ATP sulfurylases (APS1, APS3, and APS4) involved in sulfate assimilation. This was followed by the identification of phosphate deficiency induced expression of Ath-miR399 with a corresponding decrease in its target, Phosphate 2 (PHO2), a ubiquitin-conjugating enzyme (Fujii et al., 2005; Bari et al., 2006; Chiou et al., 2006). miR399 inhibits PHO2 in a dualistic manner, by promoting its transcript decay (Allen et al., 2005; Bari et al., 2006) and also by repressing translation (Bari et al., 2006).

Soil salinity has become a major limiting factor in rice production due to the salt-sensitive nature of most indica cultivars (Lutts et al., 1995; Zeng and Shannon, 2000; Moradi et al., 2003). So, understanding the biology of salt stress associated miRs holds a promising answer for identifying the channels that can reduce yield losses. It was reported that miR396, miR168, miR167, miR165, miR319, miR159, miR394, miR156, miR393, miR171, miR158, and miR169 were up-regulated in salt stress in *Arabidopsis* (Liu et al., 2008), while Ath-miR398 was down-regulated under salt stress (Jagadeeswaran et al., 2009). Similarly, salt stress in *Populus trichocarpa*, down-regulated miR530a, miR1445, miR1446a-e, miR1447, and miR171l-n but up-regulated, miR482.2 and miR1450 (Lu et al., 2008). Likewise, in *Phaseolus vulgaris*, increased accumulation of miRS1 and miR159.2 was observed in response to NaCl addition (Arenas-Huertero et al., 2009). Recently, detailed analysis of the expression levels of individual miRs or a group of miRs has improved our understanding on their role in the stressful environment. The salt stress kinetics revealed the induction of two known miRs (miR171p and miR4416d), and two novel miRs (gly_1, gly_3) in soybean root nodules (Dong et al., 2013). In rice, the time series of salt and alkali treatment was found to de-regulate the expression of Osa-miR393 and Osa-miR396 (Gao et al., 2011). Further thorough profiling showed that, of the six members in Osa-miR396 family, only Osa-miR396c was down-regulated under salt and alkali treatment (Gao et al., 2010). Osa-miR408 showed a differential expression pattern among drought susceptible and tolerant cultivars of rice, where it decreases in the sensitive cultivar but remained at elevated levels in the tolerant cultivar (Mutum et al., 2013). Such studies suggest that comprehensive expression profiling of miRs under stress is important for understanding their precise regulatory roles.

Our group has focussed on identifying miRs that affect growth and yield in rice and we have earlier reported key molecules that exhibit tissue-preferential expression patterns (Mittal et al., 2013). In this study we describe the detailed expression profile of a salt stress deregulated Osa-miR820. It is a rice specific miR, that was first reported from undifferentiated embryogenic callus tissues as miR583 (Luo et al., 2006). Osa-miR820 belongs to a family of three closely related members. These represent the canonical 21 nucleotide (nt) miRs that have been named Osa-miR820a-c and their sequences are available as MI0005263, MI0005264, MI0005265 in mirBase version 21 (Kozomara and Griffiths-Jones, 2014). Later it was proposed to be a member of the transposon derived small RNA family, with its expression being controlled epigenetically at its own locus (Nosaka et al., 2013a,b). Nosaka et al. (2013b) also identified the 24-nt length variants of this family and described two additional family members, Osa-miR820d and Osa-miR820e. Recently, it has been shown to be down-regulated under arsenic stress in two contrasting arsenic responsive rice cultivars (Sharma et al., 2015). Here, we have investigated its role in salt, high temperature and drought stress responses by comparing the expression profiles of Osa-miR820 and its target gene across various tissues of two indica rice cultivars exhibiting a contrasting response to salt stress. The cultivars used include Pusa Basmati 1 (PB1), a breeder variety of basmati which is highly sensitive to salt stress and Pokkali (PK), which is known to thrive well in highly saline soils on salty backwaters of Kerala (Rana et al., 1999; Xie et al., 2000).

## Materials and Methods

### Plant Material and Growth Conditions

Rice seeds were washed thoroughly with sterile water and placed on germinating sheets. The seeds were grown under controlled conditions at, 28 ± 2°C, 70% relative air humidity and 16/8-h light/dark cycle. For all further analysis, leaf and root tissue samples were harvested from 15 days old seedlings while floral tissues were collected at different stages from rice plants grown in controlled conditions of the green-house. Salt stress was given by replacing nutrient solution with 250 mM NaCl. Heat stress was given by keeping the plants in growth chamber at 42°C. For drought stress, rice seedlings were grown in 100 mM mannitol solution. For time kinetics each stress was provided for the time period of 30 min, 1, 3, 6, 12, and 24 h.

### RNA Isolation and Small RNA Library Preparation

Total RNA was extracted from various rice tissues using guanidine isothiocyanate (GITC) based protocol as described previously (Mittal et al., 2013). Briefly, plant tissue is homogenized in liquid nitrogen and GITC buffer is added along with phenol and chloroform. The mixture is allowed to thaw slowly and centrifuged at high speed (∼13000 rpm) for 15 min. The aqueous phase thus obtained is extracted with phenol:chloroform solution twice and kept for precipitation with ethanol. The pellet is washed using 75% DEPC-ethanol twice at 13000 rpm for 15 min each and dried at room temperature. The dried pellet of RNA was dissolved in required amount of DEPC-treated water and stored in –20°C.

For deep sequencing the small RNA libraries were constructed from normal or salt stressed leaf tissues of 15 days old seedlings as described previously (Mittal et al., 2013). Sequencing was performed using GAII sequencer, Illumina.

### Sequence Analysis of Osa-miR820 Family in Rice

Sequences of Osa-miR820 precursors were aligned with TIGR version 7 (Ouyang et al., 2007) and 93-11 genome (Zhao et al., 2004) to search all the loci of Osa-miR820 in japonica and indica sequences. Genomic locations of Osa-miR820 family members were identified by using Bowtie 1.0 (Langmead et al., 2009). Exact hits were plotted with the help of MapChart 2.3 (Voorrips, 2002) after converting the chromosome positions to base pair locations in Mbp (Khan et al., 2014). The deep sequencing data was analyzed using in house developed pipelines (Mittal et al., 2013). Digital signatures of Osa-miR820 expression for each dataset was calculated as transcripts per million (TPM) for comparisons across data sets.

### Modified 5′-RLM-RACE for Target Validation

To map the cleavage site of the target mRNA, modified 5′-RLM RACE strategy was employed using Ambion’s 5′-RLM RACE kit. Total RNA was subjected to 5′ adapter ligation using T4 RNA ligase followed by cDNA preparation using oligo(dT) primers. The polymerase chain reactions (PCRs) were performed using 5′ adapter outer forward primer: 5′-GCTGATGGCGATGAATGAACACTG-3′ and target outer reverse primer: 5′-ACCATTTCCTGCTTCCTGTGA-3′. For verification nested PCRs were performed using 5′ adapter inner primer: 5′-CGCGGATCCGAACACTGCGTTTGCTGGCTTT GATG-3’ and target inner primer: 5′-ACCAGAACCATCAGAGTGAGGA-3′. The PCR amplified band was eluted and cloned into pGEMT-easy vector. Ten such clones were sent for sequencing to map the cleavage site.

### Stem-Loop and Semi-Quantitative RT PCR

The expression levels of mature miR820 were analyzed using stem-loop end point PCR with slight modifications (Varkonyi-Gasic et al., 2007). Briefly 500 ng total RNA was used to synthesize cDNA using miR-specific stem-loop primer with Superscript reverse transcriptase III (Invitrogen) as per manufacturer’s specifications. A pulsed RT reaction was performed in a thermal cycler as follows: 30 min at 16°C, 60 cycles at 30°C for 30 s, 42°C for 30 s and 50°C for 1 s. RT enzyme was inactivated by incubating the reaction at 85°C for 5 min. 1 μl of direct cDNA was used for PCR using miR specific forward primer (5′-TCGGCCTCGTGGATGG-3′) and universal reverse primer (5′-GTGCAGGGTCCGAGGT-3′) to get a 63 bp amplification products. A 4% agarose gel was used to visualize the RT product. 18S rRNA was used as an endogenous control. Relative abundance was calculated as percentage integrated density values (%IDV) by normalizing the obtained values with 18S rRNA.

For target RT-PCR, 500 ng total RNA was used as starting material to prepare first strand cDNA using Superscript reverse transcriptase II (Invitrogen) with random hexamers as primers. 1 μl direct cDNA was used for PCR using forward primer (5′-GCGAGAAGATTATCGGACGAG-3′) and reverse primer (5′-TCGTCACCAGAACCATCAGA-3′), with 28 cycles of amplification to give 520 bp amplification products. 18S rRNA was used as endogenous control. Spot density measurements were performed using Alpha Imager software. The values were plotted after normalizing with the endogenous control.

### Statistical Analysis

All the experiments were repeated atleast three times and biological replicates were employed wherever possible. Mean of three experimental replicate values was plotted and standard deviation is shown as error bars. Paired *t*-test was performed for all the data using GraphPad Prism version 6.05 for windows (GraphPad, CA, USA). For all samples *P* < 0.05 was obtained, suggesting that the variations reported here are statistically significant.

## Results and Discussion

### Mapping miR820 on the Indica Genome

**FIGURE 1:**
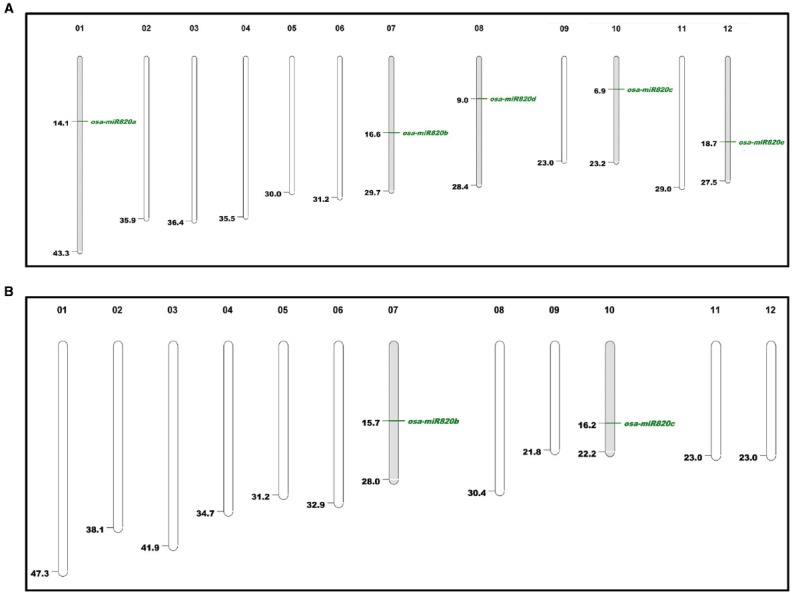
**Karyoview of Osa-miR820 family in (A) japonica and (B) indica genomes.** Chromosome number is given above each chromosome and mapping positions are shown by green lines.

The available information on Osa-miR820 family was used to search for homologs in the indica genome and the results are summarized in Table 1. As miR820 has not been mapped on the indica genome, we attempted to locate all the members on the Beijing indica 93-11 genome based on homology search. Two loci corresponding to pre-miR820b and pre-miR820c were mapped on chromosome 7 and 10, respectively (Figure 1). The locus on chromosome 1 could not be mapped. This may be attributed to the lack of full annotation of the indica genome. The pre-miR820 sequences lie in a repeat rich region of genome flanked by CACTA type DNA retrotransposons on both ends. This is in line with the postulate that young miRs generally originate by segmental duplication events mediated by transposition or recombinational events at the repeat rich regions of genome (Allen et al., 2004; Fahlgren et al., 2010; Cuperus et al., 2011). The Osa-miR820 seems specific to rice, as homologs have not been detected in other plants. The non-conserved nature of this miR also suggests its recent origin.

**TABLE 1:** **List of Osa-miR820 sequences identified in rice**.

**miR**	**Sequence**	**Size**	**Chromosome number**	**Strand**	**Location**
**Japonica**					
Osa-miR820a	TCGGCCTCGTGGATGGACCAG	21 nt	Chr01	Plus	14130483.. 14130503
	TCGGCCTCGTGGATGGACCAGGAG	24 nt	Chr01	Plus	14130483.. 14130506
Osa-miR820b	TCGGCCTCGTGGATGGACCAG	21 nt	Chr07	Minus	16576897.. 16576917
	TCGGCCTCGTGGATGGACCAGGAG	24 nt	Chr07	Minus	16576897.. 16576920
Osa-miR820c	TCGGCCTCGTGGATGGACCAG	21 nt	Chr10	Plus	6928685.. 6928705
	TCGGCCTCGTGGATGGACCAGGAG	24 nt	Chr10	Plus	6928685.. 6928708
Osa-miR820d	TCGGCCTCGTGGATAGACCAG	21 nt	Chr08	Minus	9049622.. 9049642
	TCGGCCTCGTGGATAGACCAGGAG	24 nt	Chr08	Minus	9049622.. 9049645
Osa-miR820e	TCGGCCTTCTGGATGGACCAG	21 nt	Chr12	Plus	18660106.. 18660126
	TCGGCCTTCTGGATGGACCAGGAG	24 nt	Chr12	Plus	18660106.. 18660129
**Indica**					
Osa-miR820b	TCGGCCTCGTGGATGGACCAG	21 nt	Chr07	Minus	15659779.. 15659799
	TCGGCCTCGTGGATGGACCAGGAG	24 nt	Chr07	Minus	15659779.. 15659802
Osa-miR820c	TCGGCCTCGTGGATGGACCAG	21 nt	Chr10	Minus	16204026.. 16204046
	TCGGCCTCGTGGATGGACCAGGAG	24 nt	Chr10	Minus	16204026.. 16204049

Both the indica loci contained sequences corresponding to 21-nt and 24-nt length variants of the mature miRs (Nosaka et al., 2013b). It has been established that the canonical 21-nt miRs are generated in a DCL1-dependent pathway and regulate gene expression through post transcriptional gene silencing (PTGS) involving transcript cleavage or translation repression (Vazquez et al., 2008; Zhu et al., 2008; Wu et al., 2010). The 24-nt miRs, commonly referred as long miRs, are produced through the DCL3-mediated pathway and they are supposed to be involved in regulation of gene expression via DNA methylation (Wu et al., 2010). This indicates that the Osa-miR820 family might be involved in a dual mode of target regulation.

### Digital Expression Status of Osa-miR820

**FIGURE 2:**
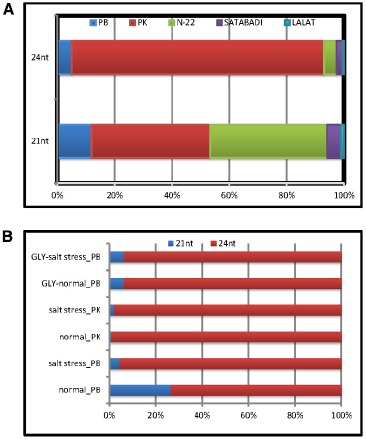
**Relative expression patterns of Osa-miR820 length variants.** The graph was plotted based on the TPM values obtained from the Illumina sequencing data of different small RNA libraries of rice. The values are presented as percentage to calculate the relative expression. **(A)** Relative expression in the healthy panicle tissues from different indicia rice cultivars. PB, Pusa Basmati 1; PK, Pokkali; Gly, glyoxalase overexpressing PB1 transgenics; N-22, Nagina 22; Satabdi, and Lalat. **(B)** Relative expression in the normal (unstressed) and salt stressed leaf tissues from different rice cultivars.

The digital expression status of Osa-miR820 family, in the leaf and panicle tissues of different indica rice cultivars, was obtained from the Illumina sequencing data available in the lab. The analysis revealed that both the length variants were present in all the libraries and the 24-nt species accumulated to a higher level as compared to the 21-nt species (Figure 2). On comparing the Osa-miR820 levels in the panicle tissues of different indica cultivars, it was observed that the relative concentration of the 21-nt species was higher in the salt-tolerant PK and the heat tolerant Nagina 22 (N22) rice cultivars followed by the salt-sensitive PB1 as compared to the dry season short-duration Satabdi and high yielding drought tolerant Lalat rice cultivars. Whereas the 24-nt species accumulated to very high levels in the PK rice cultivar (Figure 2A). This suggests that the 24-nt length variants may be regulating the genetic machinery to influence the salt-tolerant behavior in PK plants.

On examining the relative expression patterns of Osa-miR820 length variants in response to salt stress it became apparent that the 21-nt miR was down-regulated in PB1 causing a relative up-regulation of the 24-nt length variant (Figure 2B). In PK leaves the expression of 21-nt miR was up-regulated by more than five-fold but the relative expression levels of the two length variants did not change. A similar observation for Osa-miR820 was made in the salt-tolerant glyoxalase over-expressing transgenics (Singla-Pareek et al., 2008). This suggests that though the expression of Osa-miR820 is deregulated by salt stress and the ratios between the 21-nt and 24-nt length variants may be playing an important role in governing the plants response to salt stress. It also suggests a common scheme of regulation may prevail for its regulation in the salt-tolerant versus the salt-sensitive cultivars. A recent study by Mutum et al. (2013) also indicates such a regulatory layout for the Osa-miR408, which is down-regulated in a drought-susceptible variety and up-regulated in a drought-tolerant variety.

### Osa-miR820 Target Prediction and Validation

The miRs are known to regulate gene expression by interacting with their cognate targets in a highly complementary manner. To identify the transcripts that are affected due to changes in expression levels of the Osa-miR820, three different *in silico* approaches were used. The first approach involved prediction using psRNA target finder, a small RNA target analysis server developed mainly for plants (Dai and Zhao, 2011). Stringent parameters, of no mismatch between 9 and 11 nt, maximum expectation score of 3.0 and Unpaired Energy of 25, predicted seven targets (Table 2). In a second approach the targets were searched in the degradome database StarBase, using the publicly available data (Yang et al., 2011). Degradome sequencing is a high-throughput technique that provides a comprehensive means of analyzing patterns of RNA degradation. Both the techniques identified the *DNA methyltransferase* as a prominent target (Table 2). This transcript has also been reported as a target in the Zhonghua 11 rice variety (Luo et al., 2006).

**TABLE 2:** **List of predicted targets for Osa-miR820 using different approaches**.

	**Accession ID**	**Score**	**Function**
psRNA target prediction	LOC_Os03g02010.4	2	DNA cytosine methyltransferase Zmet3, putative, expressed
	LOC_Os03g02010.3	2	DNA cytosine methyltransferase Zmet3, putative, expressed
	LOC_Os03g02010.1	2	DNA cytosine methyltransferase Zmet3, putative, expressed
	LOC_Os03g02010.2	2	DNA cytosine methyltransferase Zmet3, putative, expressed
	LOC_Os11g13650.1	3	ATCSLC12, putative, expressed
	LOC_Os11g03310.1	2.5	NAC domain-containing protein 77, putative
	LOC_Os10g42196.1	3	Expressed protein
	LOC_Os02g39920.1	3.5	AT Hook family protein, putative, expressed
Degradome database search	LOC_Os05g47470	2.5	Retro-transposon protein, expressed, putative, unclassified
	LOC_Os03g02010	2.5	DNA methyltransferase protein, putative, expressed
Indica EST database analysis	CT860708	2.0	DNA (cytosine-5)-methyltransferase OsDRM2
	CK073442	3.0	Rice putative protein-coding gene
	CK046160	3.0	Putative aquaporin PIP1-5

To validate this as a target transcript in the indica genome the miR binding site was searched in the expressed sequence tags (EST) database, as its fully annotated transcriptome is unavailable (Table 2). Again the putative cytosine *DNA methyltransferase* was identified as a target transcript. Its closest rice homolog corresponds to *Domain Rearranged Methyltransferase 2 (OsDRM2*; Os03g0110800). These proteins are known to be involved in catalyzing the *de novo* methylation of the C-5 residue of cytosine along with enzyme, *Chromomethylase 3* (CMT3; Chan et al., 2005; Saze et al., 2012; Stroud et al., 2014). This role is implicated in genome defense against transposons and epigenetic activity during reproductive development and stress (Cao and Jacobsen, 2002; Sahu et al., 2013). Interestingly, the 24-nt Osa-miR820 family members were also shown to target and repress the OsDRM2 at the transcriptional level (Nosaka et al., 2013b). Thus, both 21- and 24-nt Osa-miR820 length variants seems to regulate the same target though at different points indicating that the dynamics of the plant genetic machinery may require dual mode of regulation.

The EST analysis also identified a putative *NAC domain containing protein 77* (N77) and a putative *aquaporin*, *PIP1-5*, as targets of Osa-miR820. The N77 is a member of one of the largest families of plant-specific transcription factors which have multifarious roles such as embryonic and floral development, lateral root formation, auxin signaling and stress response (Olsen et al., 2005). Whereas *PIP1-5*, also known as plasma membrane intrinsic protein 1–5, is a conserved protein, known to be involved in water channel activity and can thus affect salt stress responses by regulating transport through water channels (Marmagne et al., 2004; Gawwad et al., 2013).

**FIGURE 3:**
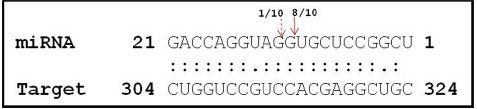
**Cleavage site mapping of Osa-miR820 target DNA cytosine-5-methyltransferase (OsDRM2).** The 5′ end of the cleaved product determined by 5′ RACE followed by cloning and sequencing. The cleavage site is indicated by an arrow in the miRNA:mRNA base-pairing diagram. The numbers indicate the clones in which the cleavage site was mapped, among the total clones analyzed.

Considering the ubiquitous presence of *DNA methyltransferase* in the prediction results, LOC_Os03g02010 was validated as a cleavage target by modified 5′-RACE strategy in both PB1 and PK cultivars (Figure 3). This indicates that the 21-nt miRs are regulating the targets at the PTGS level by target cleavage across different rice genotypes. Therefore to understand the functional significance and putative interaction of Osa-miR820 and OsDRM2 during reproductive development and abiotic stress detailed transcript profiling was performed.

### Expression of miR820 and Its Targets in Different Tissues

**FIGURE 4:**
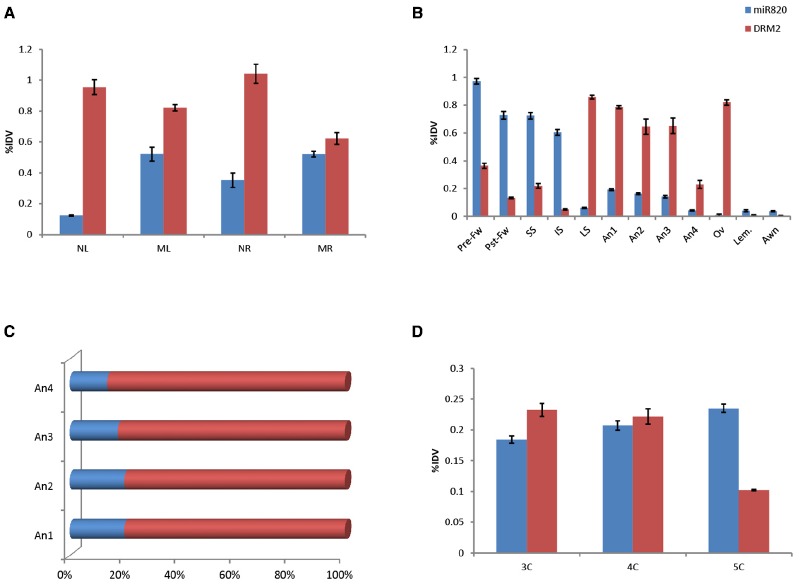
**Expression of Osa-miR820 and its target, OsDRM2 in various tissues of Pusa Basmati 1. (A)** The vegetative tissues from 15 days old seedlings and mature plant. NL, seedling leaf; ML, mature leaf; NR, seedling root; MR, mature root. **(B)** The floral tissues from late booting stage. Pre-Fw, pre-pollinated flower; Pst-Fw, post-pollinated flower; SS, superior spikelets; IS, inferior spikelets; LS, leaf sheath covering panicle; An1, anther stage 1; An2, anther stage 2; An3, anther stage 3; An4, anther stage 4; Ov, ovary; Lem, lemma. **(C)** The ratio of OsmiR820:OsDRM2 at the different stages of the anther. **(D)** Embryonic callus at different times post initiation. 3C, 3-week old callus; 4C, 4-week old callus; 5C, 5-week old callus. The integrated density values (IDV) were normalized with 18S rRNA and plotted as percentage relative abundance. The error bards indicate the standard deviation.

To delineate the role of Osa-miR820 in the physiology of rice the expression levels of the miR and OsDRM2 transcripts were checked in different tissues of PB1 and PK (Figure 4). It was observed that though Osa-miR820 is present in all the tissues, its expression was abundant in the mature leaf and root tissues (Figure 4A). The OsDRM2 levels were elevated in young leaf and root tissues, however a clear negative correlation could not be observed in the mature tissues (Figure 4A). This ambiguity may be due to overlapping expression domains of individual miR loci as the amplifications provided an overall status of the miR family due to high sequence similarity of the various Osa-miR820 isoforms. Thus it was broadly speculated that the window of transcript regulation might be restricted to the young or developing tissues of rice.

The expression levels of Osa-miR820 were also checked within the developing floral tissues (Figure 4B). It was observed that at the late booting stage when the panicles were completely encased within the flag leaf sheath (Moldenhauer and Slaton, 2001), the superior spikelets had a higher level of this miR as compared to inferior spikelets. This is in line with the earlier observations (Peng et al., 2011), and negatively correlated with the OsDRM2 transcripts. Nevertheless there was no direct correlation of the target accumulation with the fluctuations in miR expression (Figure 4B). Analysis of individual spikelets showed a two-fold higher accumulation of Osa-miR820 at pre-pollinated stage compared to the post-pollinated stage. The levels of OsDRM2 were reduced in these stages though relatively higher levels of the target transcripts were present in the pre-pollinated spikelets as compared to the post-pollinated spikelets (Figure 4B). This indicated that the miR is preferentially expressed during early reproductive developmental stage in rice and during this time the OsDRM2 transcripts were maintained at low levels.

Within the florets the ovary, lemma and awn had very low levels of Osa-miR820, though the OsDRM2 transcripts accumulated only in the ovary. Relatively higher level of Osa-miR820 was observed in the anthers at stage 1–3 representing the stage before anthesis, after anthesis and at the time of dehiscence, respectively, as compared to stage 4 representing the anthers after dehiscence. The anther stages 1–4 correspond to stage 11–14 as described earlier (Zhang et al., 2011). It was observed that within the anthers the expression of OsDRM2 was high. However, the ratio of Osa-miR820:OsDRM2 was maintained at stages 1 and 2 and decreased slowly in stages 3 and 4 (Figure 4C). This indicates the association of Osa-miR820:OsDRM2 regulation in pollen biology, which may in turn regulate the grain filling and/or grain quality in rice.

Tissue profiling was extended to the embryogenic callus tissues to check the level of miR and its target transcript (Figure 4D). Higher accumulation of both was observed in calli at 3- and 4-weeks post initiation. The reverse-correlation was captured in calli at 5 weeks post initiation, when the Osa-miR820 levels got enhanced while the OsDRM2 levels decreased. Absence of miR:target correlation at earlier stages could be due to overlapping functional domains of miR and target gene (Mittal et al., 2013). Analyzing the spatio-temporal expression of miRs is pivotal in understanding their role in plant growth and development. Recently, focused research on tissue preferential miRs has helped in understanding organ development and phase transitions in a lucid manner (Rubio-Somoza et al., 2009; Wollmann and Weigel, 2010; Mittal et al., 2013; Pandey et al., 2014). The profiling of Osa-miR820 and its target transcript in the different tissues enabled the identification of its regulatory zones. It was observed that the Osa-miR820 expression was more in the early reproductive phases and late embryogenic callus as compared to the other vegetative tissues. However, as the members of Osa-miR820 family share identical mature sequences it was difficult to predict which locus was active in these regions.

### Expression of miR820 and Its Targets in Diverse Rice Cultivars

**FIGURE 5:**
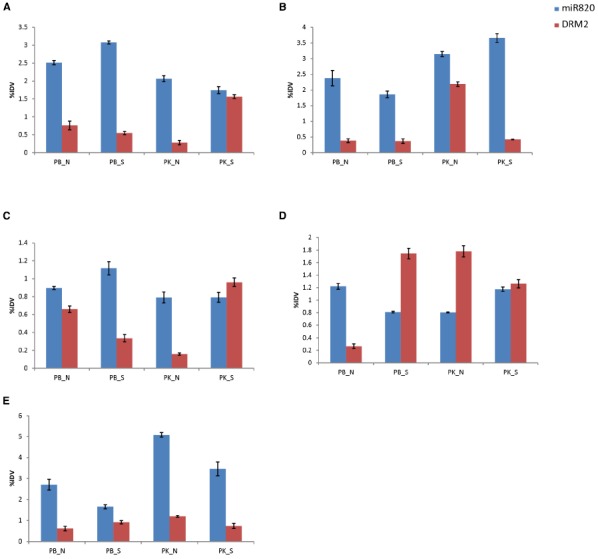
**Expression profiling of Osa-miR820 and its target, OsDRM2 in different floral tissues of Pusa Basmati 1 (PB) and Pokkali (PK) under control (N) and salt stressed (S) conditions. (A)** superior spikelets, **(B)** inferior spikelets, **(C)** pre-pollinated panicle, **(D)** post-pollinated panicle, **(E)** flag leaf. The integrated density values (IDV) were normalized with 18S rRNA and plotted as percentage relative abundance. The error bards indicate the standard deviation.

Considering the observations that Osa-miR820 is expressed during early reproductive developmental stages and is deregulated under salt stress, we investigated its expression profiles in various floral tissues under salt stress conditions, using both salt-susceptible (PB1) and salt-tolerant (PK) cultivars (Figure 5). It was observed that as compared to PB1, in PK superior spikelets accumulated lower levels while the inferior spikelets accumulated higher levels of Osa-miR820 (Figures 5A,B). A similar pattern was observed for OsDRM2 expression levels as well indicating that the 21-nt miR mediated cleavage regulation alone may not be operative in these tissues. Under salt stress, the levels of Osa-miR820 did not change extensively though a reverse pattern of deregulation was obtained in PB1 and PK. It was interesting to note that in salt stressed PK, the OsDRM2 levels decreased in the inferior spikelets but increased in the superior spikelets (Figures 5A,B). Superior spikelets flower earlier than inferior spikelets and contribute more toward grain filling as compared to inferior spikelets which either fill poorly or are sterile, which leads to compromised yield in most cases (Dong et al., 2012). This differential expression of Osa-miR820 in different zones of panicle indicates an important role of Osa-miR820 in controlling rice panicle formation.

In pre-pollinated floral tissues, salt stress induces the expression of Osa-miR820 in PB1, with corresponding decrease in OsDRM2 expression levels (Figure 5C). But in PK, a mild decrease in Osa-miR820 levels is accompanied by increase in OsDRM2 levels in salt stress. However, at the post-pollinated stage an antagonistic expression pattern is observed. In PB1, Osa-miR820 gets down-regulated leading to increase in OsDRM2 expression levels, while in PK Osa-miR820 gets up-regulated leading to decrease in OsDRM2 expression levels (Figure 5D). This indicates a delicate but controlled regulation of Osa-miR820 under changing developmental stages and time. The levels of Osa-miR820 were similar between salt stressed PB1 and normal PK tissues in pre-and post-pollinated panicles (Figures 5C,D). This indicates that gene regulatory patterns of unstressed PK cultivar are closer to salt challenged PB1 cultivar. Further understanding of this regulation is important as it could have important implications in crop improvement program for stress tolerance.

Flag leaf is the top-most leaf enclosing the emerging panicle. It also helps in assimilating a major portion of incoming solar radiations and thus, contributes to the carbohydrate synthesis for accumulation during grain filling (Li et al., 1998). The expression profiling in flag leaf revealed a greater accumulation of Osa-miR820 in flag leaf of PK as compared to PB1, though the levels declined under salt stress (Figure 5E). The OsDRM2 transcripts were maintained at lower levels though a concomitant fluctuation in its expression was not observed.

### Kinetics of miR820 and Its Target Under Salt Stress

**FIGURE 6:**
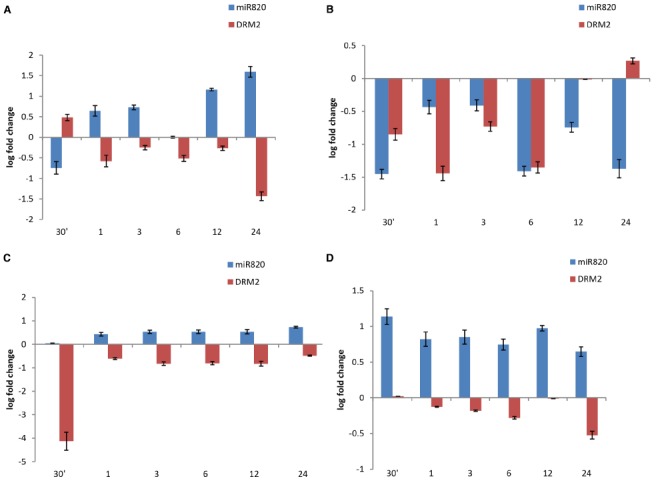
**Time kinetics of fold change of Osa-miR820 and its target OsDRM2 under salt stress in leaf and root tissues of Pusa Basmati 1 (PB) and Pokkali (PK).** The stress was given to the leaf (L) or root (R) tissues for 30 min (30′); 1 h (1); 3 h (3); 6 h (6); 12 h (12); and 24 h (24). Salt stress was provided by giving 250 mM NaCl to **(A)** leaf tissues of PB and **(B)** root tissues of PB, **(C)** leaf tissues of PK and **(D)** root tissues of PK. The error bards indicate the standard deviation.

The analysis was extended to understand the deregulation of Osa-miR820 and its target under increasing duration of salt stress, in 14-day-old seedlings of PB and PK. A time kinetics of 250 mM salt stress indicated an increase in the Osa-miR820 levels in the leaf (Figure 6A) and a decrease in the root tissues (Figure 6B). It was observed that in the leaves at 30 min of stress there is decrease in Osa-miR820 but it gradually increases by 24 h of stress (Figure 6A). A contrasting pattern was obtained for OsDRM2 indicating regulation by the miR. A similar observation on the salt stress induced deregulation of OsDRM2 transcript was reported from *Arabidopsis* seedlings (Rae et al., 2014). In both rice and *Arabidopsis* leaves, the OsDRM2 levels are induced at 30 min of stress and then decrease at 1 h although OsDRM2 is regulated by Osa-miR820 in rice only. This indicates that an overlapping and complicated regulatory network is operative in rice. In roots, low levels of Osa-miR820 were maintained throughout the stress (Figure 6B) but the levels of OsDRM2 begin to increase only at 24 h of stress period. Time course experiments for individual miRs are essential to understand the dynamism between the miR:target pair.

The time kinetics of salt stress revealed an increase in levels of Osa-miR820 in PK leaves similar to the pattern observed in PB1 though there was difference in the level of miR expression (Figure 6C). However, the miR levels were much higher in PK roots as compared to the PB1 roots (Figure 6D). The OsDRM2 transcript levels were reduced in both the tissues. In leaves, a sharp dip in OsDRM2 transcripts was observed after 30 min of stress and relatively low levels were maintained at subsequent time points (Figure 6C) whereas in roots the transcript levels decreased slowly over the stress duration of 24 h (Figure 6D). The results indicate a variation in the behavior of the miR between the salt-tolerant and susceptible varieties. Such variations have been reported earlier as with Osa-miR408 where the time kinetics of drought stress revealed its differential response in drought susceptible and tolerant rice cultivars (Mutum et al., 2013). Similarly, comparative expression analysis identified Osa-miR393 and Osa-miR396c as salt down-regulated miRs and their over-expression conferred salt sensitivity to the transgenic plants (Gao et al., 2010, 2011). These studies will contribute toward understanding the role of miRs in governing the stress responses in plants.

### Expression of miR820 and Its Targets Under Other Abiotic Stresses

**FIGURE 7:**
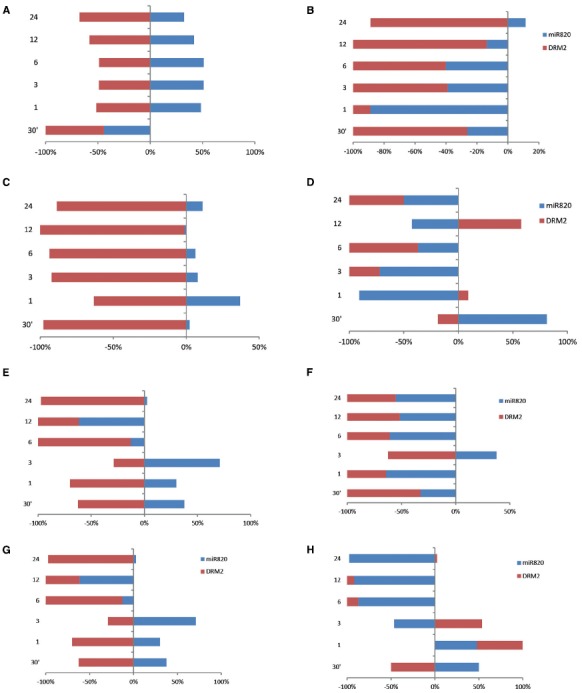
**Time kinetics of Osa-miR820 and its target OsDRM2 under high temperature and drought stress.** The stress was given to the leaf (L) or root (R) tissues of Pusa Basmati 1 (PB) and Pokkali (PK) for 30 min (30′); 1 h (1); 3 h (3); 6 h (6); 12 h (12); and 24 h (24). High temperature stress was provided by subjecting at 42°C to **(A)** leaf tissues of PB and **(B)** root tissues of PB, **(C)** leaf tissues of PK and **(D)** root tissues of PK. Physiological drought stress was given by 100 mM mannitol solution to **(E)** leaf tissues of PB and **(F)** root tissues of PB, **(G)** leaf tissues of PK and **(H)** root tissues of PK. The integrated density values (IDV) were normalized with 18S rRNA and plotted as percentage relative abundance. The error bards indicate the standard deviation.

The analysis was extended to understand the deregulation of Osa-miR820 and its target under high temperature and drought stress. The time kinetics of high temperature stress indicated an increase in the Osa-miR820 levels in the leaves of PB1 (Figure 7A) and PK (Figure 7C). The levels of OsDRM2 transcripts were correspondingly low though the extent of deregulation was more in PK as compared to PB1. In the root tissues of PB1 the expression of Osa-miR820 decreased though the concomitant increase in the levels of OsDRM2 was not observed (Figure 7B). In PK roots a high variation in the levels of Osa-miR820 and OsDRM2 was observed (Figure 7D).

In the presence of physiological drought conditions, the Osa-miR820 levels were up-regulated in the leaves till 3 h beyond which these reduced drastically. This was accompanied by a corresponding change in the OsDRM2 levels (Figures 7E,G). In PB1 roots, however, both Osa-miR820 and OsDRM2 levels were maintained at low levels under drought stress except at the 3 h time point (Figure 7F). In PK roots, Osa-miR820 levels decreased gradually after 1 h of stress but OsDRM2 transcripts accumulated till 3 h beyond which they decreased drastically (Figure 7H). The general pattern that emerged from the time kinetics studies suggests a stress induced increase in the Osa-miR820 levels in PB1 and PK leaves accompanied by a down-regulation of OsDRM2. In the root tissues both Osa-miR820 and OsDRM2 decreased in PB1 although the Osa-miR820 levels increased during early stages of stress.

## Conclusion

The results indicate that Osa-miR820 expression is precisely regulated in the different tissues of rice. In general, it accumulates more in the aerial and floral tissues as compared to the root tissues. Within the floral tissues, the miR levels were maintained at high levels in the anthers. It was observed that under salt, high temperature and drought stress the miR levels were up-regulated in the leaf tissues whereas the miR levels were down-regulated in the root tissues. A variation in the levels of miR was observed between the salt-susceptible PB1 and salt-tolerant PK varieties, but the overall pattern of deregulation appeared to be similar. The regulatory role of this miR on OsDRM2 transcripts was evident and the narrow windows of transcript regulation were captured. However, it is important to understand that the OsDRM2 transcript levels may also be influenced at the transcriptional level by the 24-nt Osa-miR820 species. Knowledge on the delicate balance in the ratios of the 21- and 24-nt miRs and their influence on the transcript expression and accumulation is required to reveal the intricate genetic reprogramming mediated by the Osa-miR820. It will also be interesting to decipher the expression zones of individual Osa-miR820 loci and understand their deregulation in stress.

### Conflict of Interest Statement

The authors declare that the research was conducted in the absence of any commercial or financial relationships that could be construed as a potential conflict of interest.
